# Editorial Comments on Immediate Repair for Laceration of the Tunica Albuginea of the Corpora Cavernosa and Penile Urethral Injury Caused by Blunt Trauma to the Flaccid Penis: A Case Report

**DOI:** 10.1002/iju5.70205

**Published:** 2026-06-01

**Authors:** Fumito Yamabe

**Affiliations:** ^1^ Department of Urology, School of Medicine, Faculty of Medicine Toho University Tokyo Japan

The authors report on a relatively rare case of blunt trauma‐induced damage to the tunica albuginea of the corpora cavernosa and its immediate repair. This is a very valuable report for the management of trauma in flaccid penises.

Generally, damage to the tunica albuginea of the corpora cavernosa of the penis occurs mainly due to excessive force applied to an erect penis. Damage to the tunica albuginea of the corpus cavernosum is rare as a result of trauma to a flaccid penis [1, 2, 3].

Although the injury did not occur during erection, damage to the tunica albuginea and urethra was promptly diagnosed. Immediate repair of these injuries resulted in a favorable outcome. The authors also examined the appropriateness of immediate reconstructive surgery in this case. In this case, although it was a compressive blunt trauma, both urethral damage and damage to the tunica albuginea of the corpora cavernosa of the penis were present, making immediate reconstructive surgery appropriate [4].

Furthermore, it is interesting that the authors consider the mechanism of tunica albuginea damage in this case to be different from typical penile fractures, and to be a compression‐type injury similar to a straddle‐type injury [4].

The important message from this report is that damage to the tunica albuginea of the corpora cavernosa can occur even in cases of non‐erect penile trauma. In cases of blunt trauma to a flaccid penis, few urologists consider the possibility of damage to the tunica albuginea, such as a penile fracture.

Considering the possibility of tunica albuginea damage in non‐erect penile trauma during examination and testing will enable prompt treatment.

While penile fracture accompanied by bloody urethral discharge should raise suspicion of urethral injury [5], conversely, when examining urethral injury due to penile trauma, damage to the tunica albuginea of the corpus cavernosum should also be suspected.

One of the problems is follow‐up after treatment.

Generally, long‐term follow‐up after treatment for penile fractures and penile trauma is often not performed due to patient‐related factors. We hope for future reports regarding long‐term issues with sexual and urinary function.

## Conflicts of Interest

The author declares no conflicts of interest.

## Data Availability

Data sharing not applicable to this article as no datasets were generated or analysed during the current study.
